# Repurposing of Ciclopirox to Overcome the Limitations of Zidovudine (Azidothymidine) against Multidrug-Resistant Gram-Negative Bacteria

**DOI:** 10.3390/pharmaceutics14030552

**Published:** 2022-03-01

**Authors:** Hyejin Cho, Kwang-sun Kim

**Affiliations:** Department of Chemistry and Chemistry Institute for Functional Materials, Pusan National University, Busan 46241, Korea; chj9512@pusan.ac.kr

**Keywords:** ciclopirox, drug repurposing, zidovudine (azidothymidine), multidrug-resistant, motility

## Abstract

Multidrug-resistant (MDR) Gram-negative bacteria are the top-priority pathogens to be eradicated. Drug repurposing (e.g., the use of non-antibiotics to treat bacterial infections) may be helpful to overcome the limitations of current antibiotics. Zidovudine (azidothymidine, AZT), a licensed oral antiviral agent, is a leading repurposed drug against MDR Gram-negative bacterial infections. However, the rapid emergence of bacterial resistance due to long-term exposure, overuse, or misuse limits its application, making it necessary to develop new alternatives. In this study, we investigated the efficacy of ciclopirox (CPX) as an alternative to AZT. The minimum inhibitory concentrations of AZT and CPX against MDR Gram-negative bacteria were determined; CPX appeared more active against β-lactamase-producing *Escherichia coli*, whereas AZT displayed no selectivity for any antibiotic-resistant strain. Motility assays revealed that β-lactamase-producing *Escherichia coli* strains were less motile in nature and more strongly affected by CPX than a parental strain. Resistance against CPX was not observed in *E. coli* even after 25 days of growth, whereas AZT resistance was observed in less than 2 days. Moreover, CPX effectively killed AZT-resistant strains with different resistance mechanisms. Our findings indicate that CPX may be utilized as an alternative or supplement to AZT-based medications to treat opportunistic Gram-negative bacterial infections.

## 1. Introduction

The emergence of multidrug-resistant (MDR) Gram-negative bacteria urgently require new strategies to treat infections caused by such bacteria. High costs and prolonged development time characterize new antibiotics. Drug repurposing, i.e., the identification of non-antibiotics for new therapeutic indications, is a promising alternative or adjuvant to treat MDR bacterial infections, obviating antibiotic-resistant biological pathways [1].

Many non-antibiotic drugs used as anticancer, antifungal, anthelmintic, and anti-inflammatory agents possess antibacterial properties [2,3,4], and ~20 such drugs have been identified as candidates for drug repurposing or as adjuvants to antibiotics against MDR Gram-negative bacteria, including *Escherichia coli* (*E. coli*) [5,6].

Zidovudine (also known as azidothymidine, AZT), an antiviral drug used for human immuno-deficiency virus (HIV) treatment [7] and commercially available as Retrovir^®^, is regarded as a leading bactericidal drug or synergistic agent to antibiotics because it shows low minimum inhibitory concentration (MIC) values against MDR Gram-negative bacteria and increases the efficacy of amikacin, gentamicin, and last-resort antibiotics [8,9,10,11,12,13,14,15]. Despite its potential action as an antibacterial agent, drawbacks, including a high mutagenic potential [16], hematologic toxicity, and association with strong resistance development in *E. coli* in short-term usage, prevent the extended use of AZT as an antibacterial agent [11,17]. Since it is still used to treat patients with HIV/AIDS, who are susceptible to opportunistic bacterial infections caused by *E. coli* [18], *Salmonella* [19], *Acinetobacter* spp. [20], *Klebsiella pneumoniae* [21,22,23], and *Pseudomonas aeruginosa* [22,23], it is necessary to find alternative agents that show similar actions on HIV and bacteria but do not have the same limitations as AZT.

Ciclopirox (CPX), a topical antifungal agent approved by United States Food and Drug Administration (FDA) in June 2004, inhibits superficial fungal infections (dermatophytosis, pityriasis, onychomycosis, and candidiasis) [24,25,26] and is considerably more effective than known agents such as itraconazole, ketonazole, and terbinafine [27]. CPX has shown an excellent safety profile and has appeared unable to promote resistance development in fungi in 31 years of use [24,28,29,30]. Oral, intravaginal, and topical administration routes for CPX have been investigated in humans, but its usage is limited as a topical agent due to poor oral or intravaginal bioavailability, gastrointestinal toxicity, and poor water solubility that limits its production as an injectable drug [31].

CPX has a completely different structure and mechanism of action compared to other known topical antifungals (azoles and allylamines) [26]. According to mechanistic studies, CPX initially acts through the chelation of polyvalent metal cations, especially iron (Fe^3+^), causing the inhibition of metal-dependent enzymes such as cytochromes, catalases, and peroxidases. Such inhibition reduces cellular activities, alters membrane permeability, disrupts DNA repair and cell division signals, disorganizes fungi mitotic spindles, and reduces the secretion of aspartyl proteinases and virulence factors necessary for *Candida albicans* infections [26]. In addition to its antifungal activity, CPX has mild anti-inflammatory effects in human polymorphonuclear cells, where CPX inhibits the synthesis of prostaglandin and leukotriene, which is mediated by scavenging reactive oxygen species (ROS) via the inhibition of 5-lipoxygenase and cyclooxygenase [24,26,32,33].

CPX has been repurposed as an anticancer [34,35,36], diabetes mellitus [37,38], antiviral—including herpes simplex virus (HSV) [39] and HIV [40,41]—and antibacterial agent [28,42,43]. In particular, CPX efficacy as an antibacterial agent has recently been highlighted as follows. CPX inhibited the growth of Gram-negative bacteria, including clinical isolates [28,42,43], and showed a synergistic activity with polymyxin B against Gram-negative bacteria [43]. Furthermore, several studies tried to understand the action mechanism of CPX as an antibacterial agent. First, genome-wide mRNA profiling showed that CPX activity can decrease the expression levels of glutamate-dependent acid resistance (GDAR) genes, such as *evgS* and *hns*, which leads to decreased motility and increased cell size [30]. Another study showed that CPX-mediated alteration of lipopolysaccharides (LPSs) stimulated enterobactin production and reduced bacterial swarming [30]. These results suggest that CPX may be used as an alternative to AZT in treating infections caused by MDR Gram-negative bacteria under AZT-mediated treatment. However, this possibility, including the efficacy and selectivity of CPX in strains with specific antibiotic resistance, has not been experimentally validated. In addition, the induction of resistance to CPX as an antibacterial agent after its long-term exposure has not been assessed.

Here, we aimed to evaluate the use of CPX as an alternative to AZT for killing Gram-negative bacteria, either control or MDR strains, by determining MIC values using a microbroth dilution method. The MIC values of AZT against five Gram-negative species ranged from 0.00625 to >6.4 µg∙mL^−1^ for control and MDR strains, with the lowest activity recorded against *A. baumannii* control strain and no difference in activity by bacterial antibiotic resistance status, except for *E. coli* and *Salmonella* species. Meanwhile, CPX was more active against β-lactamase-producing *E. coli* MDR strains. In addition, CPX killed AZT-resistant *E. coli* strains with the same efficacy as non-MDR strains. Next, the ability of CPX and AZT to induce resistance in *E. coli* cells was examined under continuous exposure to sublethal concentrations of each drug. The results demonstrated that resistance to the control *E. coli* strain did not occur after 25 d of CPX exposure, whereas AZT treatment was associated with increased resistance in the same strain by more than 1000-fold within 2 d. Our results suggest that CPX may be a potent weapon in our arsenal to compensate for the limitations of AZT against opportunistic MDR Gram-negative bacterial infections.

## 2. Materials and Methods

### 2.1. Bacterial Strains and Polymerase Chain Reaction (PCR) Amplification 

The strains used in the present study are listed in Table 1. Thymidine kinase (*tdk*) gene knockout from Keio (*E. coli* K-12 in-frame, single-gene knockout mutants) collection [44] was validated by colony PCR using the primers k1 (5′-CAGTCATAGCCGAATAGCCT) [44] and *tdk-F* [5′-GTTACCAGCAGTCATTTACCCG; −188 to −167 to the first sequence of *tdk* start codon (+1)] or the gene-specific primers *tdk*-F and *tdk*-R [5′-CGACGATGTATAACGCCTAAAC; +792 to +813 to +1] The chromosomal *tdk* gene was amplified as described above using gene-specific primers and used for sequencing. The presence of the mobilized colistin resistance (*mcr-1*) plasmid in either KS7001 or KS8001 was confirmed by colony PCR with pQE-60-specific primers [pQE-60-FW (5′-CCCGAAAAGTGCCACCTG) and pQE-60-RW (5′-GTTCTGAGGTCATTACTGG) [45]. The ASKA (A Complete Set of *E. coli* K-12 ORF Archive)-*wbbL* clone [46] was validated by colony PCR with the primers pCA24N-F (5′-GATAACAATTTCACACAGAATTCATTAAAGAG) and pCA24N-R(-*gfp*) (5′-CCCATTAACATCACCATCTAATTCAAC). All PCR reactions were carried out with Quick Taq^TM^ HS DyeMix (Cat. No. DTM-101, Toyobo, Osaka, Japan), according to the vendor’s protocol. The PCR product was identified by 1% agarose gel electrophoresis in 0.5 X TAE buffer and imaged using a ChemiDoc^TM^ MP Imaging System (Bio-Rad, Hercules, CA, USA) and Image Lab^TM^ Software (v. 5.2.1, Bio-Rad, Hercules, CA, USA).

### 2.2. Preparation of Bacterial Cells for Assays

The bacterial cells used for antibacterial activity and phenotypic characterization were prepared as follows. Initially, bacterial colonies were re-streaked on LB agar plates with or without antibiotics (kanamycin or ampicillin) and resuspended in nuclease-free water (NFW) to 0.5 McFarland turbidity using a Sensititre™ Nephelometer (Thermo Fisher Scientific, Waltham, MA, USA). Individual cells were inoculated into Sensititre™ Cation-adjusted Mueller–Hinton broth (Thermo Fisher Scientific, Waltham, MA, USA) at a 1000-fold dilution and were grown further at 37 °C for 16 h without shaking [43].

### 2.3. Evaluation of Antibacterial Activity

The minimum inhibitory concentration (MIC) for bacterial strains was determined by the microbroth dilution method on 96-well plates as described in previous reports [30,43]. Briefly, bacterial cells with 0.5 McFarland turbidity were diluted 1000-fold in Sensititre™ Cation-adjusted Mueller–Hinton broth w/TES (Cat. No. T3462, Thermo Fisher Scientific, Waltham, MA, USA) with or without zidovudine (Cat. No. PHR1292; Sigma-Aldrich, Saint Louis, MO, USA), CPX (Cat. No. C0415; Sigma-Aldrich, Saint Louis, CA, USA), or Isopropyl β-D-1-thiogalactopyranoside (IPTG) at the desired concentration. The 96-well plates were then incubated at 37 °C for 16 h, followed by imaging using a digital camera (Samsung NX200, Suwon, Korea) in triplicate experiments. Bactericidal activity was analyzed by spotting (5 µL) the onto LB-agar plates cultures in the presence of different concentrations of drugs and incubating at 37 °C for 16 h, followed by imaging with a digital camera (Samsung NX200, Suwon, Korea) in triplicate experiments.

### 2.4. Western Blotting Analysis 

The expression profile of the Mcr-1 protein from the KS8001 strain was analyzed as described in a previous report [45] using an Anti-His tag Antibody (Cat. No. 105327-MM02T, Sino Biological, Wayne, PA, USA) and Clarity™ Western ECL substrate (Bio-Rad, Hercules, CA, USA). The blots were imaged using a ChemiDoc^TM^ MP Imaging System (Bio-Rad, Hercules, CA, USA) and Image Lab^TM^ Software (ver 5.2.1, Bio-Rad, Hercules, CA, USA). One representative image from three independent experiments (*n* = 3) is shown.

### 2.5. DNA Sequencing and Sequence Alignment

DNA sequencing of the *tdk* gene product from *E. coli* ATCC 25922, AZT-R, and BW25113 strains using *tdk* gene specific primers (*tdk*-F and *tdk*-R) was performed using the chain termination method and an automatic sequencer by BioFact (Daejeon, Korea) with the same primers for sequencing. Sequence alignment of *tdk* genes from *E. coli* strains was performed using Clustal Omega (ClustalW2, v2.1, http://www.clustal.org; accessed on 10 January 2022) [50].

### 2.6. Cell Morphology Analysis

To identify the increase in cell length, scanning electron microscopy (SEM) image analysis by Scanning Electron Microscopy (SEM, TESCAN, Fuveau, France) was performed. Bacterial cells were cultured with or without CPX at 37 °C, while shaking at 500 rpm. Morphological analysis of the cells was performed as described [45].

### 2.7. Motility Assay

Swimming motility was determined as previously described, with minor modifications [30]. Solid-agar plates for swimming motility were incubated at 37 °C for 6 h and imaged using a ChemiDoc^TM^ MP Imaging System (Bio-Rad, Hercules, CA, USA) and Image Lab^TM^ Software (ver 5.2.1, Bio-Rad, Hercules, CA, USA). The experiments were performed in triplicate, and colony size was measured using a transparent ruler.

### 2.8. Generation of Laboratory-Made Drug-Resistant E. coli Strains

AZT-and CPX-resistant *E. coli* strains were generated as follows. Initially, ATCC 25922 cells (10^6^ CFU∙mL^−1^) were incubated with sublethal concentrations (1/2 MIC) of AZT (1.6 µg∙mL^−1^) and CPX (12.5 µg∙mL^−1^) and cultured for 16 h at 37 °C. Aliquots from the resulting cultures were inoculated, as described above, to produce bacteria resistant to AZT and CPX. This process was repeated for 15 and 25 d, respectively. The MIC values of AZT and CPX for drug-treated ATCC 25922 cells in individual passages were measured to determine the sublethal concentration and the fold increase in resistance compared to non-treated ATCC 25922 cells. Generation of tigecycline (TIG)-resistant ATCC 19606 strain was carried out with the method reported above. For this purpose, TIG (Cat No. 1667643, Sigma-Aldrich, Saint Louis, MO, USA) was used. The most resistant colony to TIG was defined as *A. baumannii* TIG-R 19606 (Figure S1).

### 2.9. Statistical Analysis

Statistical analysis was performed using GraphPad Prism 8 (GraphPad Software, San Diego, CA, USA). All data were obtained from at least three biological replicates and are presented as mean value ± standard deviation.

## 3. Results and Discussion

### 3.1. Activity of AZT and CPX against Gram-Negative Species with Different Antibiotic Resistance Status

Our initial aim was to compare the efficacy of AZT and CPX in the same target species with different antibiotic resistance status; thus, we intended to select bacterial species as study targets known to be susceptible to both AZT and CPX. AZT was found to have significant bactericidal activity against *Enterobacteriaceae* (*E. coli*, *K. pneumoniae*, and *S. typhimurium*), but no activity against *P. aeruginosa* and Gram-positive bacteria [8]. Among them, *A. baumannii* and extended-spectrum β-lactamase (ESBL)-producing strains including *Enterobacteriaceae* (*E. coli*, *K. pneumoniae*, and *S. typhimurium*) have been identified as threatening opportunistic bacterial pathogens in patients and are at the top of the Centers for Disease Control and Prevention (CDC) priority list [18,19,20,51]. Therefore, four species known to be susceptible to both drugs were selected as targets for assessing the activity of AZT and CPX. Additionally, control or MDR (or clinical isolates) *A. baumannii*, *E. coli*, *K. pneumoniae*, and *S. typhimurium* strains with different antibiotic resistance status, including to cell-wall-degrading antibiotics or last-resort antibiotics (tigecycline or colistin), were selected to evaluate the activity of AZT or CPX on them. First, the efficacy of AZT and CPX against control strains of *A. baumannii*, *E. coli*, *K. pneumoniae*, and *S. typhimurium* was determined by MIC values. As shown (Table 1 and Figures S2 and S3), the MIC values of AZT for the *E. coli* strains ranged from 0.00625 to 0.8 µg∙mL^−1^. In addition, AZT MIC for control *A. baumannii* (ATCC 17978 and ATCC 19606), *K. pneumoniae* (KCTC 1726), and *S. typhimurium* 14028S strains was >6.4, 1.6, or 0.4 µg∙mL^−1^, respectively (Table 1). This indicates that AZT was more active against *E. coli* and *Salmonella* than against *A. baumannii* and *K. pneumoniae*. Meanwhile, the MIC of CPX for *A. baumannii*, *E. coli*, and *S. typhimurium* was 25 µg∙mL^−1^, whereas it was 100 µg∙mL^−1^ for *K. pneumoniae* (Table 1), indicating that the *K. pneumoniae* strain is not a good target for CPX among the tested strains.

Interestingly, one of the control strains, BW25113, was more susceptible to AZT than ATCC 25922. Unlike ATCC 25922, a clinical isolate producing a smooth LPS (serotype 6) [52], BW25113 did not produce the O-antigen of LPS (rough LPS) due to an IS*5* insertion in the *wbbL* gene involved in O-antigen synthesis [53]. This similar phenomenon was identified in another K-12 *E. coli* strain (MG1655) (Table 1), which did not produce O-antigens [53]. Therefore, we hypothesized that the substantial increase in susceptibility observed for BW25113 could be attributed to the absence of the O-antigen in the LPS structure. 

To test the above hypothesis, we determined AZT susceptibility as the MIC value for an *E. coli* BW25113 strain overexpressing *wbbL* from the ASKA clone [46] to see if the susceptibility was restored to the MIC of ATCC 25922 by the expression of the O-antigen. The results showed that (Table 1; Figure S2) the MIC of AZT for the *wbbL*-overexpressing strain by IPTG induction was restored to 0.8 µg∙mL^−1^, indicating that the O-antigen in LPS is important for AZT activity. Since there the role of the LPS structure in AZT antibacterial action is not known, we additionally investigated the role of the core LPS structure on AZT activity. To this end, AZT activity against the ClearColi^®^ BL21 (DE3) strain, an LPS-free *E. coli* strain [48], was evaluated to determine the MIC value. As shown in Table 1, susceptibility of ClearColi^®^ BL21 (DE3) to AZT was recorded at 0.05 µg∙mL^−1^, indicating that AZT was 10-fold less active than in BW25113 and suggesting that the LPS structure was not the main reason of BW25113′s higher susceptibility to AZT. Another explanation involved the gene *tdk*, which codes for thymidine kinase (Tdk), an enzyme that performs the first phosphorylation of AZT converting it to the active form [8,54], which might be absent or modified in the ATCC 25922 chromosome. This possibility was tested by PCR amplification of the *tdk* gene in both strains. It was found that seven nucleotides positions, i.e., at +386, +401, +513, +531, +564, +571, and +578, were mutated in ATCC 25922, according to DNA sequence alignment (Figure S4a), resulting in the change of two amino acids (Q191E and D193G) (Figure S4b). However, these changes were regarded as neutral mutations that did not affect Tdk function [55]. Therefore, our findings suggest that unidentified factors enhancing AZT susceptibility, other than LPS structure and *tdk* gene expression, may be present in the BW25113 strain. High-throughput omics studies will be required in the near future to identify such factors, which can then be used to develop novel strategies to improve AZT antibacterial activity against Gram-negative bacteria.

**Figure 1:**
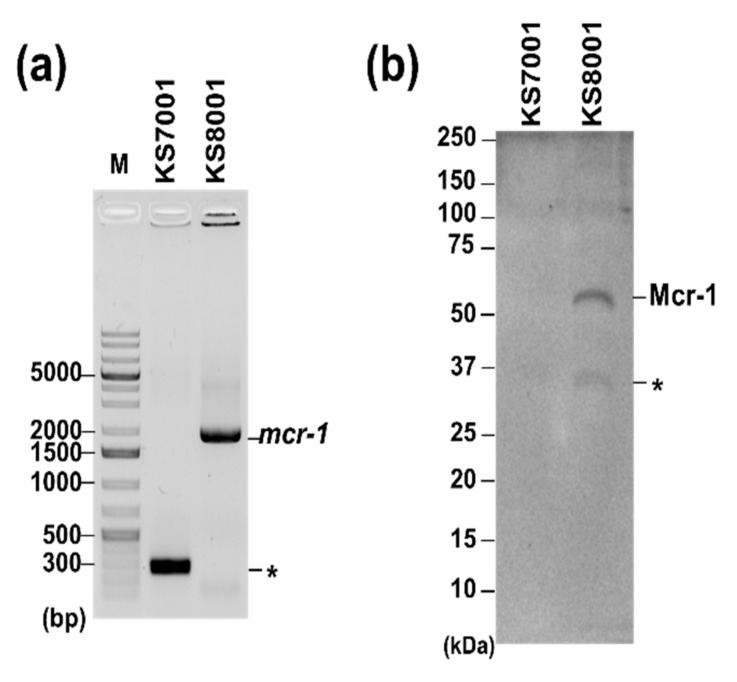
Confirmation of Mcr-1 expression in *E. coli* ATCC 25922. (**a**) Confirmation of *mcr-1* expression from the related plasmid. PCR amplification using pQE-60-specific primer sets was performed for KS7001 (pQE-60, lane 1) and KS8001 (pQE-60-*mcr-1*, lane 2), which overproduced a His-tagged Mcr-1 protein [45], on 1% agarose gel wis shown. M and asterisk (*) indicate the DNA size marker (GeneRuler 1kb Plus DNA ladder; Thermo Scientific, MA, USA) and a non-specific PCR product, respectively. (**b**) Detection of Mcr-1 protein. Mcr-1 protein from KS8001 was detected by Western blotting with an Anti-His tag Antibody (Sino Biological, Wayne, PA, USA). Image acquisition and quantitative analysis were performed by using ChemiDoc^TM^ MP Imaging System (Bio-Rad, Hercules, CA, USA) and Image Lab^TM^ Software (ver 5.2.1; Bio-Rad, Hercules, CA, USA).

Next, the effects of AZT and CPX on MDR strains with different resistance status were compared. MDR strains producing either β-lactamase or Mcr-1 were used for this purpose. First, the MICs of AZT and CPX for the laboratory available β-lactamase-expressing *E. coli*, *K. pneumoniae*, and *S. typhimurium* strains (ATCC BAAs, CCARM, and KCTC) were analyzed. The results (Table 1; Figure S2) indicated AZT MICs ranging between 0.025 and 1.6 µg∙mL^−1^, which were similar to those for *E. coli* ATCC 25922, *K. pneumoniae* KCTC 1726, and *Salmonella* 14028S strains. However, the MIC of CPX was 12.5 µg∙mL^−1^ for 62.5% of β-lactamase-expressing *E. coli* strains (CCARM 0291, 1013, 1120, 1368, ATCC BAA-2340, -2452, -2469, and -2472), lower compared to the MIC for ATCC 25922, which was 25 µg∙mL^−1^. The MICs for *Salmonella* strains (CCARM 0293, 8170, 8250, and 8254) were 25–50 µg∙mL^−1^, either the same as or 2-fold higher than that for the 14028S strain. Furthermore, the MICs for *K. pneumoniae* strains (KCTC 22507, 22058, 22062, and 32203) were 100 µg∙mL^−1^, i.e., the same as that for the control strain (KCTC 1726). This indicated that CPX was more active against β-lactamase-expressing *E. coli* strains. Second, the efficacy of AZT and CPX on last-resort antibiotic-resistant strains, including *E. coli* (colistin; NCCP 16283 and NCCP 16284) and *A. baumannii* (tigecycline; TIG-R *A. baumannii*), was evaluated. The results (Table 1; Figures S2 and S3) indicated that the MICs for AZT and CPX were the same as those for the control strains (ATCC 25922 or ATCC 19606). All the above data indicate that CPX was more active against β-lactamase-expressing *E. coli* strains than against Mcr-1-expressing *E. coli*, while there was no difference in the activity of AZT in relation to any antibiotic resistance type. Recent studies reported that AZT showed a good activity against *mcr-1*-mediated colistin-resistant strains [12,15], which is not in agreement with our data. Since *mcr-1* is a well-known component of the colistin resistance phenotype [56], ATCC 25922 cells harboring an Mcr-1-expressing plasmid (KS8001) were used to understand whether *mcr-1* itself, rather than various genetic backgrounds and antibacterial resistance statuses, was responsible for our observation (Figure 1a); therefore, the effect of *mcr-1* on AZT MIC values was further analyzed. The results (Figure S2) showed that the MIC (0.8 µg∙mL^−1^) was the same as that for the parental cells without Mcr-1 expression (KS7001), as indicated by western blotting using cellular lysates of KS8001 (Figure 1b). Therefore, Mcr-1 in *E. coli* expressing it cannot be the specific target of AZT, and other uncharacterized genetic factors are likely involved. This needs to be studied in the future.

### 3.2. Phenotypic Studies of Increased CPX Action against β-Lactamase-Expressing Resistant E. coli Strains 

Since it is known that treatment with CPX or modulation of the expression of its associated target genes leads to increased cell length and decreased motility as phenotypes [30], these phenotypes in BAA strains expressing β-lactamase were assessed. First, cell length was measured by scanning electron microscopy (SEM) (Figure S5). To this end, cells from ATCC 25922, BAA-2340, BAA-2452, and BAA-2469 strains were cultured with vigorous shaking with or without CPX at 6.25 µg∙mL^−1^, a sub-MIC concentration for BAA strains, for 3 h and imaged as described in Section 2.6. The results showed that there was no increase in cell length, as evaluated in SEM images (Figure S5). Swimming motility on a solid agar plate using the same strains was also analyzed. Initially, the motility of all strains without CPX was analyzed by measuring the diameter of the bacterial colonies on the plate. The data showed that the resistant strains were less motile than ATCC 25922 (Table 2; Figure S6). Then, motility was measured in the presence of CPX at 6.25 µg∙mL^−1^, and all strains showed a dramatic decrease in motility compared to ATCC 25922. Based on the above findings, we believe that the enhanced CPX activity is correlated with a significant reduction of motility in β-lactamase-producing MDR *E*. *coli* strains through the expressional change of motility gene(s). However, this phenotype does not appear to be related to the known CPX action targets such as *evgS* and *hns*, since CPX-mediated down-regulation of these genes both increased cell length and decreased motility [30]. Therefore, further high-throughput studies are required to identify new motility-associated genes directly involved in the enhanced susceptibility of β-lactamase-producing MDR *E*. *coli* strains.

The swimming motility of control and β-lactamase-producing MDR *E.* *coli* strains was analyzed with or without CPX at a sub-lethal concentration (6.25 μg·mL^−1^). The diameter of grown bacterial colonies on the plates was measured by a transparent ruler, and averaged values with standard deviation from triplicate experiments are shown. 

### 3.3. CPX Exhibits Bactericidal Activity against Tdk-Dependent AZT-Resistant E. coli 

It has been known that the major limitation of AZT as a repurposed antibacterial agent is the recurrence of resistance in multiple generations mainly due to the loss of Tdk activity [17,43]. Therefore, the repeated or widespread use of AZT as a repurposed drug against Gram-negative bacteria, especially *E. coli*, and associated therapies will expand difficult-to-treat bacterial populations. Since CPX usage is similar to that of AZT for its anticancer, antiviral, and antibacterial activities [2,57,58,59], it is necessary to test whether CPX eliminates AZT-resistant bacteria. For this purpose, *tdk*-knockout *E. coli* (Keio-*tdk*) [44], which lacks thymidine kinase (Tdk) expression, was used as a model of AZT-resistant strain. The MIC of AZT for Keio-*tdk* was determined and compared to that for the parental strain BW25113. As shown in Figure S2 and Table 1, the MIC for Keio-*tdk* (50 µg∙mL^−1^) was 1000 times higher than that for BW25113 (0.00625 µg∙mL^−1^), indicating that the lack of thymidine kinase (Tdk) expression was the main resistance mechanism, as previously reported [55,60]. Next, the MIC of CPX for Keio-*tdk* was assessed and compared with that for BW25113. The results showed that the MIC value of CPX for both strains was the same (25 µg∙mL^−1^), and no strong increase in the MIC was recorded, in contrast to what observed in the case of AZT. The bactericidal properties of CPX against AZT-resistant *E. coli* are reported in Figure S7. These data indicated that CPX can efficiently kill *tdk*-knockout cells, which are regarded as *tdk*-dependent AZT-resistant cells. 

### 3.4. Induction of Resistance by AZT and CPX

**Figure 2:**
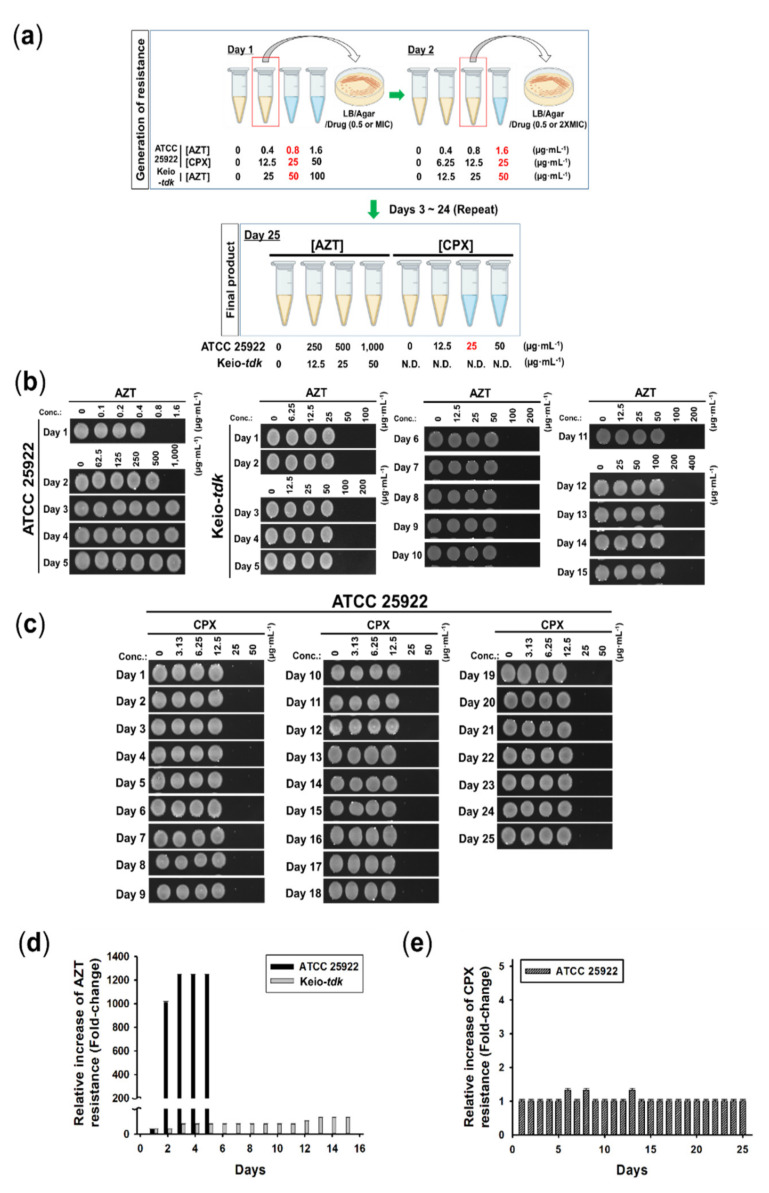
Generation of resistance phenotypes by AZT or CPX. (**a**) Schematic representation of the resistance generation method. Bactericidal activity of (**b**) AZT or (**c**) CPX against *E. coli* ATCC 25922. Representative data from *n* = 3 experiments, as described in Section 3.4 are shown. Relative increase of resistance of ATCC 25922 to (**d**) AZT or (**e**) CPX. The relative increase of resistance as a fold change with respect to non-drug-treated ATCC 25922 or Keio-*tdk* cells (set to 1 for each set) was calculated based on MIC changes from (**b**,**c**). The values shown in the graph at the indicated days are averaged values from *n* = 3 experiments, with standard deviations (*p* < 0.05).

A recent report suggested that resistance to AZT under its own pressure could occur in an unmutated-Tdk strain that contained eight mutated genes, including *rclA*, a gene associated with nucleotide binding, and a gene encoding a hypothetical protein [55]. However, the generation rate of Tdk-dependent and -independent AZT-resistant *E. coli* has not been assessed. Since the generation of AZT-resistant *E. coli* cells is widespread [55], how rapidly AZT pressure could generate resistance in the above pathways needs to be tested and compared with the rate of generation of CPX resistance. To this end, AZT- or CPX-resistant *E. coli* cells were generated with multiple passages under increasing sub-MIC concentrations of the drugs using ATCC 25922 or Keio-*tdk* cells, as depicted (Figure 2a). As shown in Figure 2b, strongly AZT-resistant *E. coli* from ATCC 25922 cells (here named AZT-R *E. coli*) were generated within 2 d with an AZT MIC >1000-fold higher than that of control cells and >20-fold higher than that of Keio-*tdk* (Figure 2; Table 1). The exact MIC of AZT higher than 1000 µg∙mL^−1^ could not be determined, since the maximum concentration of the stock solution of AZT was 10 mg∙mL^−1^. The resistance to AZT according to its MIC value was stable after 15 d of resistance generation (Figure 2b,c). However, AZT-resistant *E. coli* from Keio-*tdk* (here named AZT-*tdk*-R *E. coli*) firstly appeared after 3 d of exposure to sublethal concentration of AZT (25 µg∙mL^−1^), with only a 2-fold increase of MIC (100 µg∙mL^−1^) compared to the MIC for Keio-*tdk*. In addition, the MIC value did not changed after 15 d of continued exposure to a sub-MIC concentration (50 µg∙mL^−1^) of AZT (Figure 2b,c). This indicates that *E. coli* reduced the expression or activity of Tdk, the main mechanism to survive under AZT pressure. Therefore, AZT-mediated antiviral therapy combined with an inhibitor of Tdk expression or activity would help to reduce the severe growth of strongly AZT-resistant *E. coli* (>1000 µg∙mL^−1^ of MIC) populations. However, this strategy appears unsuitable for an AZT-based antiviral therapy, since Tdk inhibitors such as (±)-9-{[(Z)-2-(hydroxymethyl)cyclohexyl] methyl}-guanine (L-653, 180) would also reduce the antiviral potential of AZT [61].

**Figure 3:**
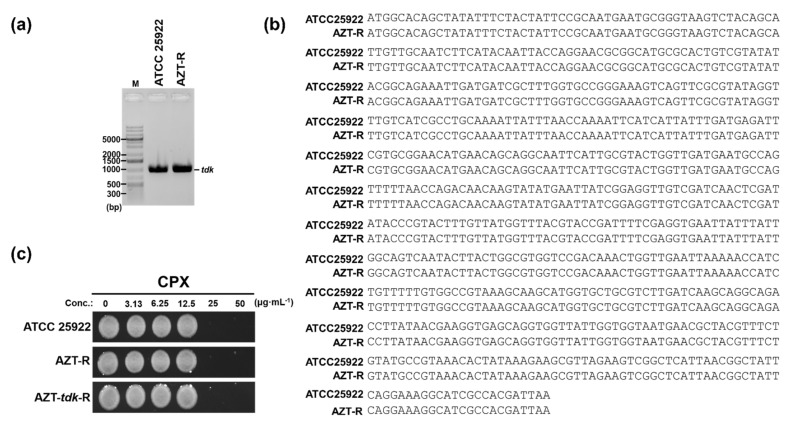
Bactericidal activity of CPX in AZT resistant strains. (**a**) PCR amplification of the *tdk*-encoding gene. Sequence information for ATCC 25922 was obtained from the NCBI database (accession No. CP009072.1). Sequence alignment of (**b**) the *tdk* gene. DNA sequencing results of *tdk*-encoding region from AZT-R and ATCC 25922, read using the primers Keio-*tdk*-F and -R, were aligned using Clustal Omega (ClustalW2, v2.1, http://www.clustal.org; accessed on 10 January 2022) [50]. (**c**) Bactericidal activity of CPX. The bactericidal activity of CPX in AZT-resistant *E. coli* cells (AZT-R or AZT-*tdk*-R) and control cells (ATCC 25922) was determined at the indicated concentrations. Data shown here are from one representative experiment from triplicate experiments. The LB agar plates were imaged with a digital camera (Samsung NX200, Suwon, Korea).

Meanwhile, CPX resistance did not generate any resistant strains when applied for 25 d (Figure 2d,e). This was the first evidence that CPX does not generate resistant populations as an antibacterial agent. Since the main target of AZT in *E. coli* is a thymidine kinase (Tdk) expressed from the *tdk* gene in the bacterial chromosome [54], we examined whether any mutation in the Tdk coding region was present. To test this possibility, PCR amplification of the *tdk* gene in AZT-R and ATCC 25922 cells was performed with *tdk*-specific primers (Figure 3a), and the products were sequenced. As shown by the alignment of the sequencing results (Figure 3b), the *tdk* gene in AZT-R cells was not mutated, suggesting that other unidentified factors contributing to AZT resistance might be presented. Therefore, AZT-R *E. coli* originated from a Tdk-independent resistance pathway, likely due to the use of AZT. The above data firstly showed that the generation of Tdk-independent AZT-resistant *E. coli* was much faster than that through of Tdk-dependent resistant cells. Moreover, CPX would not generate resistant populations when used as an antibacterial agent against *E. coli*, as shown in the antifungal profiles [26,43]. Next, the possible usage of CPX to kill AZT-resistant *E. coli* strains was evaluated. The results of the MIC assays (Figure S2) showed that the MIC of CPX for AZT-R cells was the same (25 µg∙mL^−1^) as that for the control strain, with retained bactericidal properties of CPX (Figure S2). All the above data indicate that CPX can kill any type of AZT-resistant strains. Therefore, the use of CPX to kill AZT-resistant strains appears to be feasible.

## 4. Conclusions

In summary, we showed that CPX is a potential alternative agent to AZT against Gram-negative bacterial infections, especially against *E. coli* species, which are associated with HIV/AIDS. Additionally, CPX effectively killed AZT-resistant strains characterized by different resistance mechanisms and showed higher activity against β-lactamase-producing *E. coli* strains, which are less motile in nature, and whose motility was reduced by CPX more strongly than that of a parental strain. Furthermore, CPX did not induce bacterial resistance, even after 25 d of passage, unlike AZT which rapidly induced resistance. Therefore, CPX could be an effective repurposed drug to kill both AZT- and β-lactamase-mediated resistant *E. coli*, by itself or in combination with other repurposed antibacterial drugs. However, several critical developments are required to employ CPX as an alternate antibacterial agent to AZT, due to its limitations. First, new synergistic formulations must be identified to improve the antibacterial activity of CPX to the activity level of current antibiotics. Candidates are FDA-approved compounds that are generally regarded as safe (GRAS) and share target pathways with CPX. Second, the activation of CPX action against specific bacterial species is required. The formulation of CPX into biocompatible nanoparticles is a promising strategy for this purpose, since nanoparticles have been utilized to increase the selectivity pressure of antibiotics to target species. In addition, synthetic derivatives of CPX can be utilized to increase its specificity. This possibility was proved in a recent study, in which synthesized CPX derivatives effectively inhibited the growth of *Cryptococcus neoformans* [62]. It would be worth to assess such chemicals for antibacterial activity against specific bacteria with different antibiotic resistance statuses. Third, a strategy for the rapid and complete absorption of CPX from the gastrointestinal (GI) tract following the oral route administration must be established. One potential scenario to overcome the above issue is utilizing a CPX-prodrug (Fosciclopirox or CPX-POM) [63], a novel anticancer agent currently being evaluated in patients to avoid dose-limiting GI toxicity and first-pass effect [64]. With the above developments, the clinical utility of CPX, topically or systematically administered, for eradicating opportunistic infections due to bacteria, virus, and fungi will be enhanced. 

## Figures and Tables

**Table 1:** Minimum inhibitory concentrations (MICs) of test antibiotics against Gram-negative bacterial strains.

Species	Strain	Genetic Feature or Antibiotic Resistance	MIC (μg∙mL^−1^) ^1^		Reference
			AZT	CPX	
*A. baumannii*	ATCC 17978	*Acinetobacter baumannii* Bouvet and Grimont; Control	>12.8	25	ATCC
	ATCC 19606	*Acinetobacter baumannii* Bouvet and Grimont; Control	>12.8	25	ATCC
	TIG-R	TIG^R^ ATCC 19606	>12.8	25	ATCC
*E. coli*	BW25113	K-12 *F*^−^*Δ(araD-araB)567 ΔlacZ4787*::*rrnB-3 LAM*^−^ *rph-1 Δ(rhaD-rhaB)568 hsdR514*; O-antigen (−); control	0.00625	25	[44]
	ATCC 25922	Smooth LPS (serotype 6); control	0.8	25	ATCC
	MG1655	K-12 *F*^−^ *λ*^−^*ilvG* ^−^ *rfb-50 rph-1;* O-antigen (−); control	0.00625	25	[47]
	KS7001	ATCC 25922 pQE-60 (AMP^R^)	0.8	25	This study
	KS8001	ATCC 25922 pQE-60-*mcr-1* (AMP^R^)	0.8	25	This study
	KS9000	BW25113 pCA24N(*-gfp*)	0.00625	25	[46]; This study
	KS9001	BW25113 ASKA-*wbbL*	0.00625	25	[46]; This study
	ClearColi^®^ BL21 (DE3)	*msbA148 ΔgutQ ΔkdsD ΔlpxL ΔlpxM ΔpagP ΔlpxP Δept*; LPS-free (Lipid IV_A_)	0.05	25	[48]
	Keio-*tdk*	BW25113 *tdk*::KAN^R^	50	25	[44]
	CCARM 0291	NAL	0.4	12.5	CCARM
	CCARM 1013	AMP, CEP, GM, NOR	0.2	25	CCARM
	CCARM 1120	*bla_ESBL_*; AMP, CEP, CIP, GM, NOR	0.1	25	CCARM
	CCARM 1368	AMP, CEP, CTX, GM, NOR	0.4	12.5	CCARM
	ATCC BAA-2340	*bla*_NDM-1_, AMC, AMP, TIC, PIP, CEP, CIP, CTX, FEP, FOX, DOR, MER, ETP, IMP, NAL, MOX, NOR, TOB, TET, TRI/SXT	0.8	12.5	ATCC
	ATCC BAA-2452	*bla_NDM-1_*, ETP, IMP	0.2	12.5	ATCC
	ATCC BAA-2469	*bla_NDM-1_*, ETP, IMP	0.2	12.5	ATCC
	ATCC BAA-2471	*bla_NDM-1_*, ETP, IMP	0.05	25	ATCC
	NCCP 16283	*mcr-1*, AMP, CAZ, CHL, CIP, COL, FEP, FOX, GEN, NAL, SXT, TET	0.8–1.6	25	NCCP
	NCCP 16284	*mcr-1*, *bla_NDM-1_*; *bla_TEM-1_*, *bla_CTX-M-27_*, AMC, AMP, CAZ, CHL, CIP, COL, DOR, ETP, FEP, FOX, IMP, MEM, NAL, SXT, TET	0.05	25	NCCP
	AZT-R	Tdk-independent AZT^R^ from ATCC 25922	>1000	25	This study
	AZT-*tdk*-R	Tdk-dependent AZT^R^ from Keio-*tdk*	200	25	This study
*K. pneumoniae*	KCTC 1726	*Klebsiella pneumoniae* subsp. *pneumoniae*; Control	1.6	100	KCTC
	KCTC 22057	*Klebsiella* sp.; clinical isolate	1.6	100	KCTC
	KCTC 22058	*Klebsiella pneumoniae*; clinical isolate	1.6	100	KCTC
	KCTC 22062	*Klebsiella* sp.; clinical isolate	1.6	100	KCTC
	KCTC 32203	*Klebsiella pneumoniae*; clinical isolate	1.6	100	KCTC
*S. typhimurium*	14028S	*Salmonella enterica* serovar *typhimurium*; Control	0.4	25	[49]
	CCARM 0293	*bla*_AmpC_; AMP, NAL	0.4	25	CCARM
	CCARM 8170	*bla_AmpC_*; AMP, CHL, NAL, STR, TET	0.1	50	CCARM
	CCARM 8250	*bla_AmpC_*; AMP, CHL, TET	0.1	50	CCARM
	CCARM 8254	*bla_AmpC_*; AMP, CHL, TET	0.1	50	CCARM

Abbreviations: AMC: Amoxicillin/Clavulanate (2:1), AMP: Ampicillin; AmpC: ampicillinase C; AZT: Zidovudine, CAZ: Ceftazidime, Bla: β-lactamase; CEP: Cephalothin, CIP: Ciprofloxacin, COL: Colistin, CTX: Cefotaxime, CHL: Chloramphenicol, DOR: Doripenem, ETP: Ertapenem, FEP: Cefepime, FOX: Cefoxitin, GM: Gentamicin, IMP: Imipenem, KAN: Kanamycin, MER: Meropenem, MOX: Moxifloxacin, NAL: Nalidixic acid, NOR: Norfloxacin, PIP: Piperacillin, STR: Streptomycin, SXT: Sulfamethoxazole-Trimethoprim, TET: Tetracycline, TIC: Ticarcillin, TIG: Tigecycline, TOB: Tobramycin, TRI: Trimethoprim, NDM: New Delhi metallo-β-lactamase-1, *mcr-1*: mobilized colistin resistance. ATCC: American Type Culture Collection (www.atcc.org; accessed on 10 January 2022), CCARM: Culture Collection of Antimicrobial Resistant Microbes (http://knrrc.swu.ac.kr/index.jsp; accessed on 10 January 2022), NCCP: National Culture Collection for Pathogens (https://nccp.kdca.go.kr/main.do; accessed on 10 January 2022), and KCTC: Korean Collections for Type Culture (https://kctc.kribb.re.kr; accessed on 10 January 2022). ^1^ MIC values shown are one representative from *n* = 3.

**Table 2:** Motility of *E. coli* strains under CPX treatment.

Strain	Diameter of Grown Bacterial Cells, mm	
	CPX(−) ^1^	CPX(+) ^2^
ATCC 25922 (Control)	24.0 ± 1.4	15.3 ± 2.1
ATCC BAA-2340	17.2 ± 2.1	1.33 ± 0.5
ATCC BAA-2452	15.0 ± 1.6	2.00 ± 0.8
ATCC BAA-2469	17.0 ± 2.2	2.67 ± 0.5

^1,2^ (−) and (+) denote the non-treated and treated by CPX at the sub-lethal concentration, respectively.

## Data Availability

The data presented in this study are available from the corresponding author upon reasonable request.

## Supplementary Materials

The following supporting information can be downloaded at: https://www.mdpi.com/article/10.3390/pharmaceutics14030552/s1, Figure S1: Generation of resistance phenotype by tigecycline (TIG); Figure S2: MIC determination of AZT and CPX against *E. coli* strains; Figure S3: MIC determination of AZT and CPX against *A. Baumannii*, *Klebsiella sp.*, and *Salmonella typhimurium* strains; Figure S4: Sequence alignment of Tdk encoding genes from *E. coli* ATCC 25922 and BW25113 strains; Figure S5: Morphological analysis; Figure S6: Motility assays; Figure S7: AZT and CPX activity against *tdk* knockout *E. coli* cells.
