# Supplementary material for: Repurposing of Ciclopirox to Overcome the Limitations of Zidovudine (Azidothymidine) against Multidrug-Resistant Gram-Negative Bacteria
Source: Pharmaceutics. 2022 Mar 1;14(3):552. doi: 10.3390/pharmaceutics14030552 (PMC8950944; doi:10.3390/pharmaceutics14030552)
Supplement: Supplementary file 1 [file pharmaceutics-14-00552-s001.zip › pharmaceutics-1585666-supplementary.pdf]

pharmaceutics

MDPI

Supplementary Materials: Repurposing of Ciclopirox to Overcome the Limitations of Zidovudine (Azidothymidine) against Multidrug-Resistant Gram-negative Bacteria

Hyejin Cho and Kwang-sun Kim

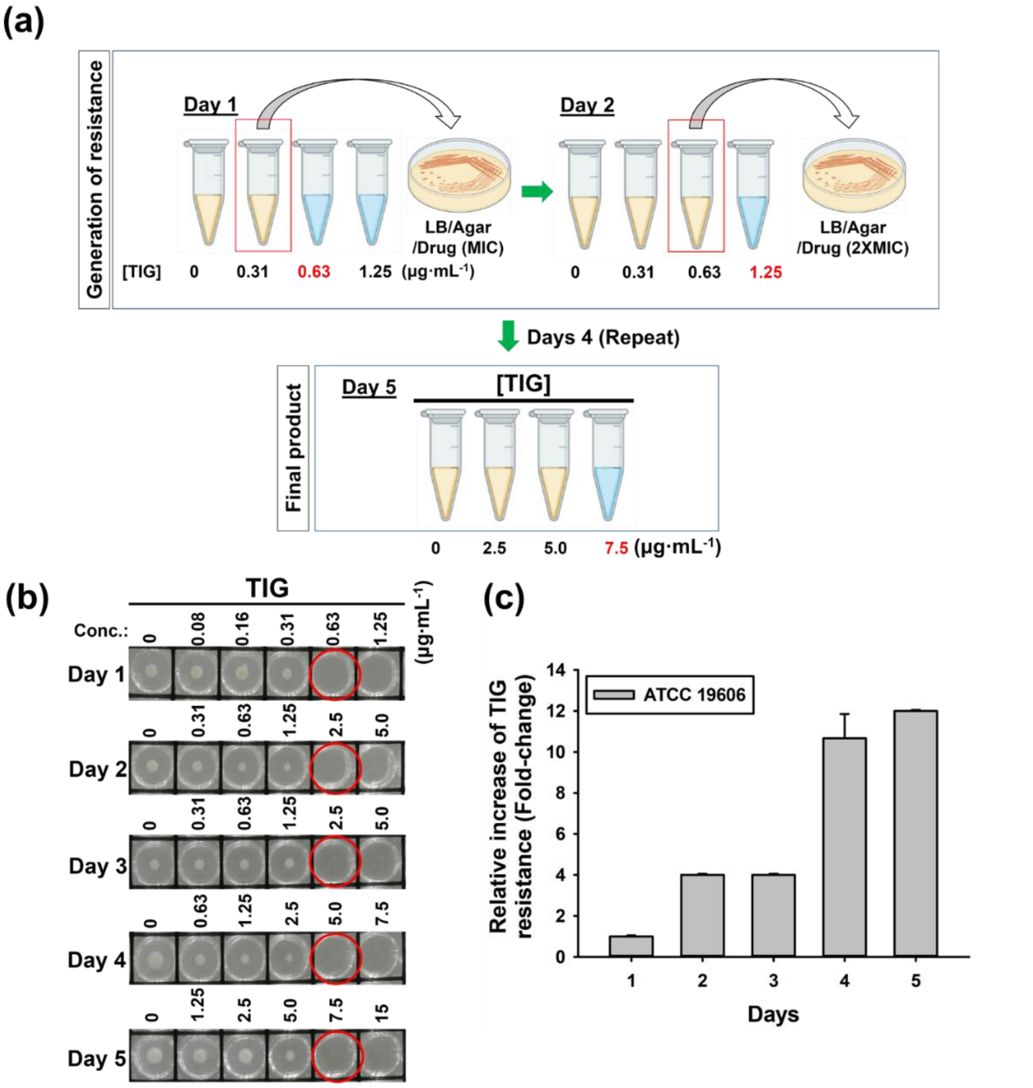

Figure S1. Generation of resistance phenotype by tigecycline (TIG). (**a**) Schematic representation of resistance generation method. (**b**) MIC values of TIG. One of the representative data from $n = 3$ was shown. (**c**) Relative increase of TIG resistance. The relative increase of resistance as a fold change to non-drug treated ATCC 25922 cells (set to 1) were calculated based on MIC changes from (**b**). The values shown in the graph at indicated days were averaged values from $n = 3$ with standard deviation ($p < 0.05$). The 96-well plates were imaged with digital camera (Samsung NX200, Suwon, Korea).

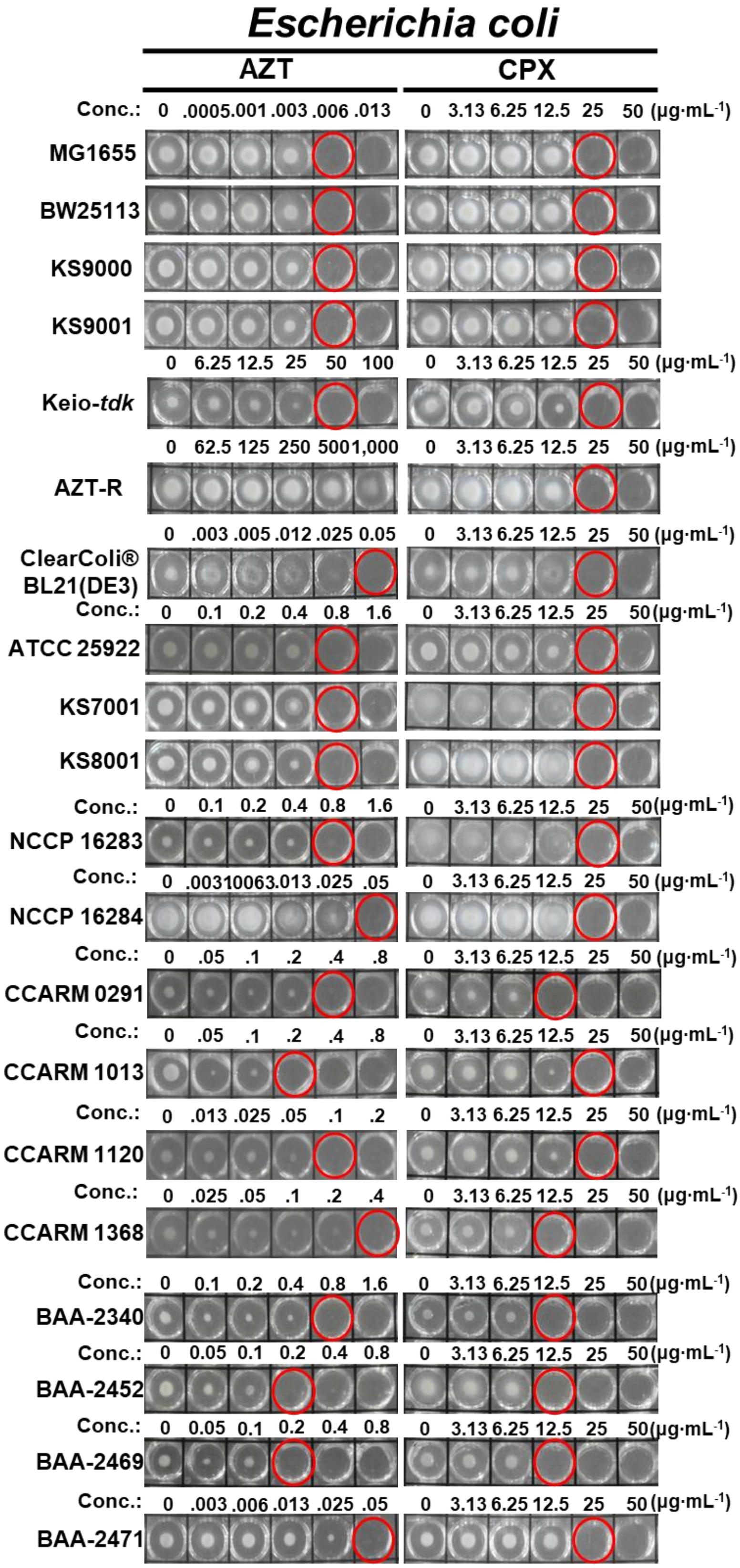

Figure S2. MIC determination of AZT and CPX against *E. coli* strains. MIC for individual strains was indicated by red circle. One of the representative data from $n = 3$ was shown. The 96-well plates were imaged with digital camera (Samsung NX200, Suwon, Korea).

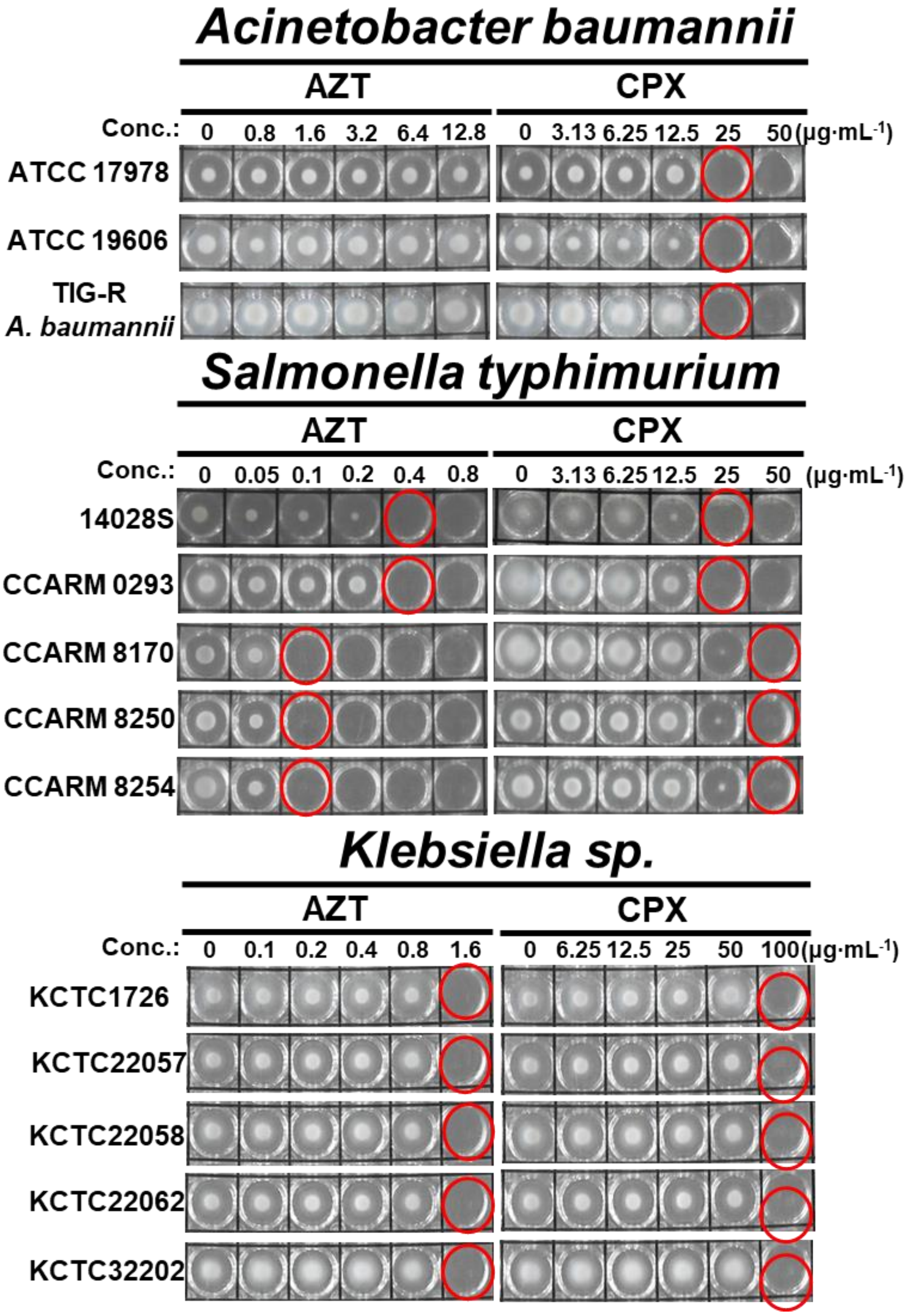

Figure S3. MIC determination of AZT and CPX against *A. Baumannii*, *Klebsiella sp.*, and *Salmonella typhimurium* strains. MIC for individual strains was indicated by red circle. One of the representative data from $n = 3$ was shown. The 96-well plates were imaged with digital camera (Samsung NX200, Suwon, Korea).

(a)

```
ATCC25922 ATGGCACAGCTATATTTCTACTATTCCGCAATGAATGCGGGTAAGTCTACAGCATTG
  BW25113 ATGGCACAGCTATATTTCTACTATTCCGCAATGAATGCGGGTAAGTCTACAGCATTG

ATCC25922 TTGCAATCTTCATACAATTACCAGGAACGCGGCATGCGCACTGTCGTATATACGGCA
  BW25113 TTGCAATCTTCATACAATTACCAGGAACGCGGCATGCGCACTGTCGTATATACGGCA

ATCC25922 GAAATTGATGATCGCTTTGGTGCCGGGAAAGTCAGTTCGCGTATAGGTTTGTCATCG
  BW25113 GAAATTGATGATCGCTTTGGTGCCGGGAAAGTCAGTTCGCGTATAGGTTTGTCATCG

ATCC25922 CCTGCAAAATTATTTAACCAAAATTCATCATTATTTGATGAGATTCGTGCGGAACAT
  BW25113 CCTGCAAAATTATTTAACCAAAATTCATCATTATTTGATGAGATTCGTGCGGAACAT

ATCC25922 GAACAGCAGGCAATTCATTGCGTACTGGTTGATGAATGCCAGTTTTTAACCAGACAA
  BW25113 GAACAGCAGGCAATTCATTGCGTACTGGTTGATGAATGCCAGTTTTTAACCAGACAA

ATCC25922 CAAGTATATGAATTATCGGAGGTTGTCGATCAACTCGATATACCCGTACTTTGTTAT
  BW25113 CAAGTATATGAATTATCGGAGGTTGTCGATCAACTCGATATACCCGTACTTTGTTAT

ATCC25922 GGTTTACGTACCGATTTTCGAGGTGAATTATTTATTGGCAGTCAATACTTACTGGCG
  BW25113 GGTTTACGTACCGATTTTCGAGGTGAATTATTTATTGGCAGCCAATACTTACTGGCA

ATCC25922 TGGTCCGACAAACTGGTTGAATTAAAAACCATCTGTTTTTGTGGCCGTAAAGCAAGC
  BW25113 TGGTCCGACAAACTGGTTGAATTAAAAACCATCTGTTTTTGTGGCCGTAAAGCAAGC

ATCC25922 ATGGTGCTGCGTCTTGATCAAGCAGGCAGACCTTATAACGAAGGTGAGCAGGTGGTT
  BW25113 ATGGTGCTGCGTCTTGATCAAGCAGGCAGACCTTATAACGAAGGTGAGCAGGTGGTA

ATCC25922 ATTGGTGGTAATGAACGCTACGTTTCTGTATGCCGTAAACACTATAAAGAAGCGTTA
  BW25113 ATTGGTGGTAATGAACGATACGTTTCTGTATGCCGTAAACACTATAAAGAGGCGTTA

ATCC25922 GAAGTCGGCTCATTAACGGCTATTCAGGAAAGGCATCGCCACGATTAA
  BW25113 CAAGTCGACTCATTAACGGCTATTCAGGAAAGGCATCGCCACGATTAA
```

(b)

```
ATCC25922 MAQLYFYYSAMNAGKSTALLQSSYNYQERGMRTVVYTAEIDDRFGAGKVSSRIGLSS
  BW25113 MAQLYFYYSAMNAGKSTALLQSSYNYQERGMRTVVYTAEIDDRFGAGKVSSRIGLSS
ATCC25922 PAKLFNQNSSLFDEIRAEHEQQAIHCVLVDECQFLTRQQVYELSEVVDQLDIPVLCY
  BW25113 PAKLFNQNSSLFDEIRAEHEQQAIHCVLVDECQFLTRQQVYELSEVVDQLDIPVLCY
ATCC25922 GLRTDFRGELFIGSQYLLAWSDKLVELKTICFCGRKASMVLRLDQAGRPYNEGEQVV
  BW25113 GLRTDFRGELFIGSQYLLAWSDKLVELKTICFCGRKASMVLRLDQAGRPYNEGEQVV
ATCC25922 IGGNERYVSVCRKHYKEALEVGSLTAIQERHRHD*
  BW25113 IGGNERYVSVCRKHYKEALQVDSLTAIQERHRHD*
```

Figure S4. Sequence alignment of Tdk encoding genes from *E. coli* ATCC 25922 and BW25113 strains. Results of (**a**) DNA or (**b**) amino acid sequence alignments of ATCC 25922 and BW25113 strains using Clustal Omega (ClustalW2, v2.1, http://www.clustal.org; accessed on 1 March 2022). Nucleotides in red were indicated different positions in two strains.

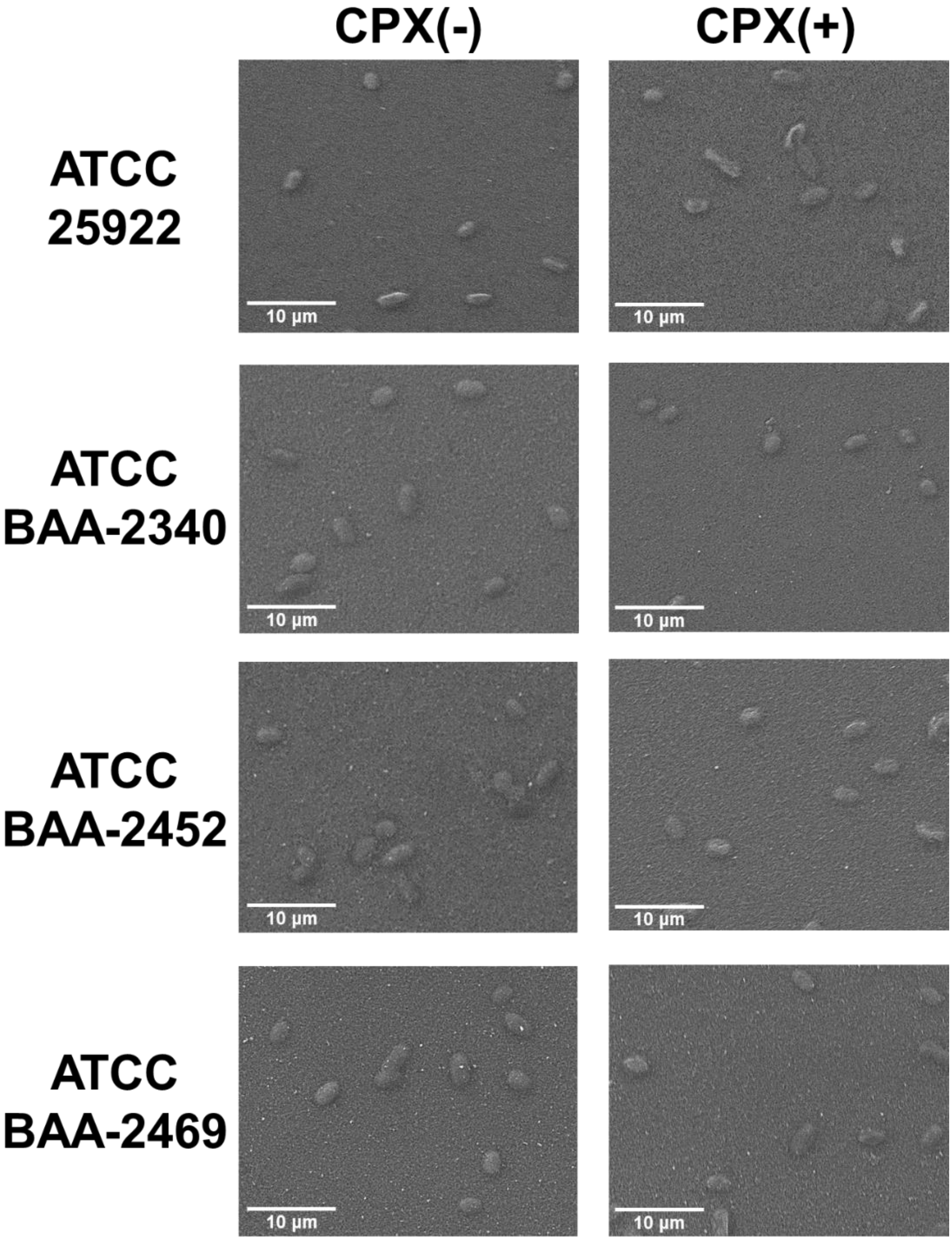

Figure S5. Morphological analysis. *E. coli* ATCC25922, ATCC BAA-2340, -2452, and -2469 cells with (+) or without (−) CPX at a sublethal concentration (6.25 µg· mL^{-1}). Scanning Electron Microscopy (SEM) images. The same cells were imaged using Scanning Electron Microscopy (TESCAN, Fuveau, France). One representative from n = 3 was shown.

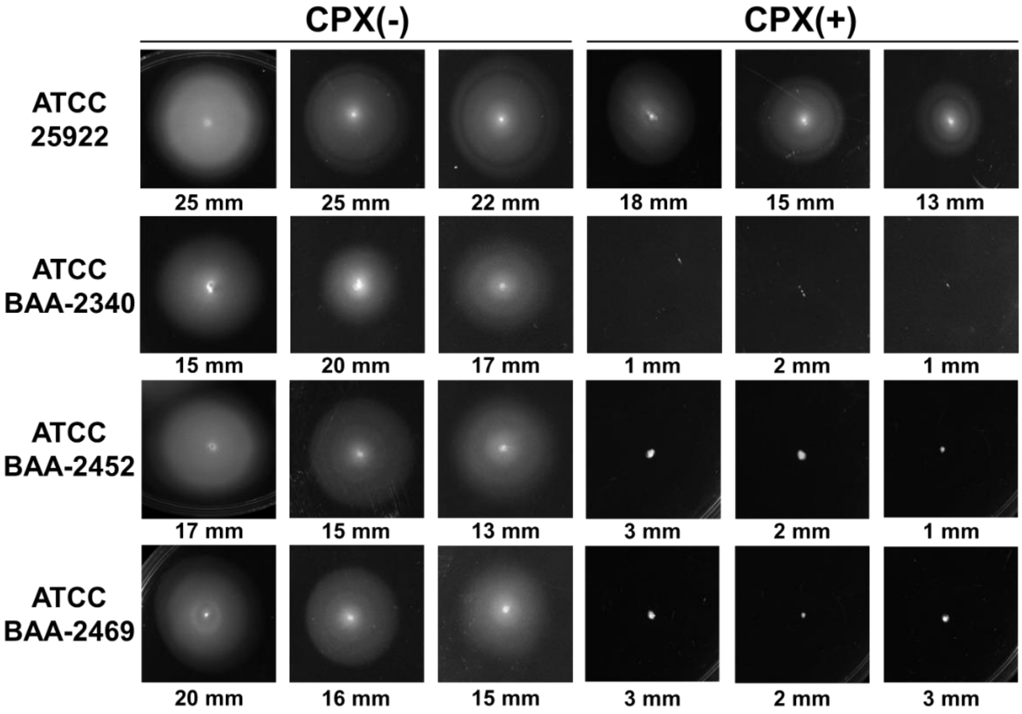

Figure S6. Motility assays. Swimming motility of *E. coli* ATCC 25922, ATCC BAA-2340, -2452, and -2469 cells with (+) or without (−) CPX at a sublethal concentration (6.25 μg·mL^{-1}) was analyzed. Images of agar plates from triplicate experiments were captured by Image Lab™ Software (ver 5.2.1; Bio-Rad, Hercules, CA, USA) and averaged values with standard deviations were shown in Table 2. The diameter of grown bacterial cells on the plates were measured by a transparent ruler and was indicated.

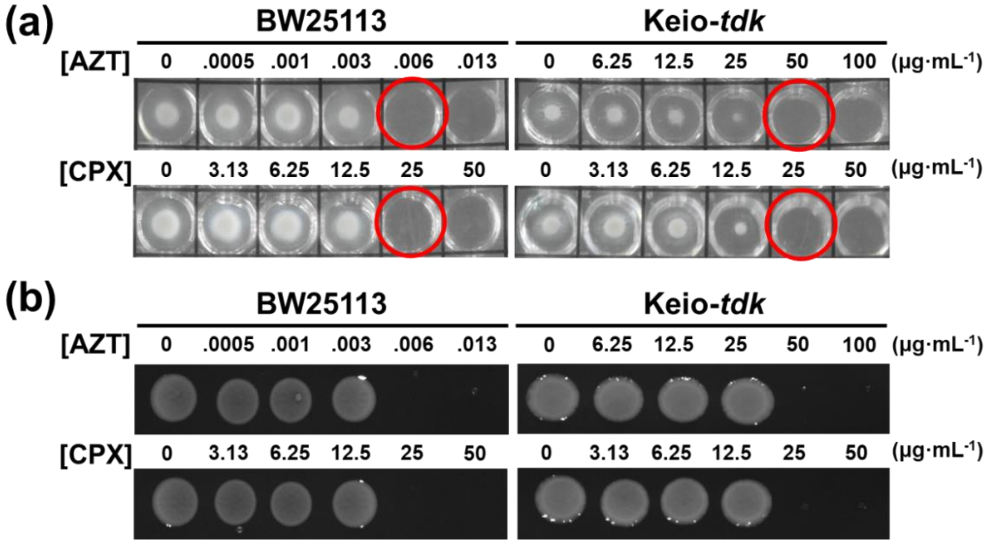

Figure S7. AZT and CPX activity against *tdk* knockout *E. coli* cells. (**a**) Determination of MIC and (**b**) bactericidal activity of AZT and CPX against *tdk* knockout (Keio-*tdk*) cells (Table 1). BW25113 was used as a control strain. Data shown here is one representative from *n* = 3. The 96-well and LB agar plates were imaged with digital camera (Samsung NX200, Suwon, Korea). One representative from triplicate experiments was shown. Red circles indicate MIC value for individual drugs.
